# Quality of life among tuberculosis (TB), TB retreatment and/or TB-HIV co-infected primary public health care patients in three districts in South Africa

**DOI:** 10.1186/1477-7525-10-77

**Published:** 2012-06-28

**Authors:** Julia Louw, Karl Peltzer, Pamela Naidoo, Gladys Matseke, Gugu Mchunu, Bomkazi Tutshana

**Affiliations:** 1 HIV/STI and TB (HAST) Research Programme, Human Sciences Research Council, Pretoria, Durban and Cape Town, South Africa; 2 Department of Psychology, University of the Free State, Bloemfontein, South Africa; 3 Department of Psychology, University of the Western Cape, Cape Town, South Africa

**Keywords:** Social functioning, Tuberculosis patients, HIV and AIDS, Quality of life, South Africa

## Abstract

**Introduction:**

TB and HIV co-morbidity amount to a massive burden on healthcare systems in many countries. This study investigates health related quality of life among tuberculosis (TB), TB retreatment and TB-HIV co-infected public primary health care patients in three districts in South Africa.

**Methods:**

A cross sectional study was conducted among 4900 TB patients who were in the first month of anti-TB treatment in primary public health care clinics in three districts in South Africa. Quality of life was assessed using the social functioning (SF)-12 Health Survey through face to face interviews. Associations of physical health (Physical health Component Summary = PCS) and mental health (Mental health Component Summary = MCS) were identified using logistic regression analyses.

**Results:**

The overall physical and mental health scores were 42.5 and 40.7, respectively. Emotional role, general health and bodily pain had the lowest sub-scale scores, while energy and fatigue and mental health had the highest domain scores. Independent Kruskal–Wallis tests found significant positive effects of being TB-HIV co-infected on the domains of mental health functioning, emotional role, energy and fatigue, social function and physical role, while significant negative effects were observed on general health, bodily pain and physical function. In multivariable analysis higher educational, lower psychological distress, having fewer chronic conditions and being HIV negative were significantly positively associated with PCS, and low poverty, low psychological distress and being HIV positive were positively significantly associated with MCS.

**Conclusion:**

TB and HIV weaken patients’ physical functioning and impair their quality of life**.** It is imperative that TB control programmes at public health clinics design strategies to improve the quality of health of TB and HIV co-infected patients.

## Introduction

Tuberculosis (TB) and HIV co-infection comprise an enormous burden on health care systems, particularly in heavily infected countries such as South Africa (SA) [1,2]. SA alone has 28% of the world’s population of HIV and TB co-infected individuals [2]. Particularly physical and mental distress is found to be common in TB patients leading to poor disease outcome or poor treatment outcome [3]. Physical health was found to be more affected than mental health when comparing between people with active TB in China and the general population [4]. In the same study using the Social Functioning (SF)-36 scale, significantly lower scores were found on Physical Function (PF), Physical Role (RP), General Health (GH), Bodily Pain (BP) and Vitality (VT) subscales and no significant differences on Emotional Role (RE), Social Function (SF) and Mental Health (MH) subscales [4]. In a study conducted among people living with HIV and AIDS the mean physical summary scores of 49.0 and mean mental summary scores of 45.6 were found using the Social Functioning questionnaire (SF-12) [5]. Significantly higher physical health and mental health summary scores were reported for patients in Uganda who completed eight months TB therapy compared to patients who only started their treatment [3]. Further, another study found that active TB patients reported lower scores across all SF-36 subscales compared to those individuals not infected by TB [6]. In addition, lower mean scores were reported on all domains of the SF-36 when comparing people with active TB to people with latent TB infection [7].

A number of factors have been identified as being associated with better health-related quality of life in tuberculosis and/or HIV patients including younger age [3,8], higher household income [8], low stigma [9], low depression and family support [1,10]. Even though there has been an increasing interest in the area of health-related quality of life (HRQoL), there still seems to be a lack of research especially among TB and HIV co-infected patients. This study investigates health related quality of life among tuberculosis (TB), TB retreatment and/or TB-HIV co-infected public primary health care patients in three districts in South Africa.

## Method

### Study design

This is a cross-sectional study among tuberculosis patients in public primary care clinics in SA.

### Study participants and sampling procedures

Three provinces in SA with the highest TB caseload were selected for inclusion in the study. One district in each province with the highest TB caseloads was ultimately included. The National Department of Health (NDoH) determined the districts with the highest TB caseloads based on District Health Information System (DHIS) information. These districts were Siyanda in the Northern Cape Province, Nelson Mandela Metro in the Eastern Cape Province and eThekwini in KwaZulu-Natal Province. Within each of these three study districts 14 public primary health care facilities were selected on the basis of the highest TB caseloads per clinic (N = 42). This was established through record review of TB cases in all clinics of the study districts over a six months period prior to the study. The type of health facilities were primary health care clinic or community health centre. All new TB and new retreatment patients were consecutively interviewed within one month of anti-tuberculosis treatment. The interviews were conducted by trained external research assistants for a period of 6 months from mid April to mid October in all 42 clinics in 2011. A health care provider who identified a new TB treatment or retreatment patient (within one month on TB treatment) of 18 years or older informed the patient about the study and referred the patient for participation if interested. This was irrespective if the patient was co-infected with HIV or not. A research assistant asked for permission/consent from patients attending the primary care facility to participate in the interview. Ethical approval from the Human Sciences Research Council Research Ethics Committee (Protocol REC No.1/16/02/11) was received. The Department of Health in South Africa has also provided approval for this study.

### Measures

#### Socioeconomic characteristics

A researcher-designed questionnaire was used to record information on participants’ age, gender, educational level, marital status, income, employment status, dwelling characteristics and residential status. *Poverty* was assessed with 5 items on the availability or non-availability of shelter, fuel or electricity, clean water, food and cash income in the past week. Response options ranged from 1 = “Not one day” to 4 = “Every day of the week”. Poverty was defined as higher scores on non-availability of essential items. The total score ranged from 5 to 20 with 20 indicative of the ‘highest level of poverty’. The categories of poverty, therefore, are as follows: 5 = low, 6–12 = medium and 13–20 = high. Cronbach alpha for the poverty index in this study was 0.89.

The 10-item *Kessler Psychological Distress Scale* (K-10) was used to measure global psychological distress, including significant pathology which does not meet formal criteria for a psychiatric illness [11,12]. This scale measures the following symptoms over the preceding 30 days by asking: “In the past 30 days, how often did you feel: nervous; so nervous that nothing could calm you down; hopeless; restless or fidgety; so restless that you could not sit still; depressed; that everything was an effort; so sad that nothing could cheer you up; worthless; tired out for no good reason?” The frequency with which each of these items was experienced was recorded using a five-point Likert scale ranging from “none of the time” to “all the time”. This score was then summed with increasing scores reflecting an increasing degree of psychological distress. The K-10 has been shown to capture variability related to non-specific depression, anxiety and substance abuse, but does not measure suicidality or psychoses [13]. This scale serves to identify individuals who are likely to meet formal definitions for anxiety and/or depressive disorders, as well as to identify individuals with sub-clinical illness who may not meet formal definitions for a specific disorder [11]. This scale is increasingly used in population mental health research and has been validated in multiple settings [14] including HIV positive individuals in South Africa [15]. There was significant agreement between the K-10 and the MINI-defined depressive and anxiety disorders. A receiver operating characteristic (ROC) curve analysis indicated that the K-10 showed agreeable sensitivity and specificity in detecting depression (area under the ROC curve, 0.77), generalized anxiety disorder (0.78), and posttraumatic stress disorder (PTSD) (0.77) [15]. Further, the K10 demonstrated moderate discriminating ability in detecting depression and anxiety disorders in the general population in South Africa; evidenced by area under the receiver operating curves of 0.73 and 0.72 respectively, with a cut off of 16 [16]. We examined the K-10 scale used as a binary variable, comparing scores of 10–15 versus 16 or more. The internal reliability coefficient for the K-10 in this study was alpha = 0.92.

#### Alcohol consumption

The 10-item Alcohol Disorder Identification Test (AUDIT) [17] assesses alcohol consumption level (3 items), symptoms of alcohol dependence (3 items), and problems associated with alcohol use (4 items). Heavy episodic drinking is defined as the consumption of six standard drinks (10 g alcohol) or more on a single occasion [17]. In South Africa a standard drink is 12 g alcohol. Because AUDIT is reported to be less sensitive at identifying risk drinking in women [18], the cut-off points of binge drinking for women (4 units) were reduced by one unit as compared with men (5 units), as recommended by [18]. Responses to items on the AUDIT are rated on a 4-point Likert scale from 0 to 4, for a maximum score of 40 points. Higher AUDIT scores indicate more severe levels of risk; scores 8 indicate a tendency to problematic drinking. The Alcohol Use Disorders Identification Test (AUDIT) was developed by the World Health Organization as an effective screening instrument for alcohol use problems among patients seeking primary care for other medical problems in international settings including African countries (Kenya and Zimbabwe) [17,19] and has been validated in HIV patients in South Africa showing excellent sensitivity and specificity in detecting MINI-defined dependence/abuse (area under the receiver-operating characteristic curve, 0.96) [20] and among TB and HIV patients in primary care in Zambia demonstrating good discriminatory ability in detecting MINI-defined current AUDs (AUDIT = 0.98 for women and 0.75 for men) [21]. Cronbach alpha for the AUDIT in this sample was 0.92, indicating excellent reliability. Hazardous drinking is defined as a quantity or pattern of alcohol consumption that places patients at risk for adverse health events, while harmful drinking is defined as alcohol consumption that results in adverse events (e.g., physical or psychological harm) [22].

#### Tobacco use

Two questions were asked about the use of tobacco products. (a) Do you currently use one or more of the following tobacco products (cigarettes, snuff, chewing tobacco, cigars, etc.)? Response options were ‘yes’ or ‘no’. (b) In the past month, how often have you used one or more of the following tobacco products (cigarettes, snuff, chewing tobacco, cigars, etc.) Response options were once or twice, weekly, almost daily and daily.

### Social functioning (SF-12)

The social functioning (SF)-12 is a measure of general health functioning. The 12 items reflect the following eight sub-domains: self-perceived general health (1 item); bodily pain (1 item); physical functioning (2 items); physical role (2 items); vitality (1 item); general health (1 item); social functioning (1 item); mental health (2 items) and emotional role (2 items); Cronbach alpha was 0.80 in this study. For each respondent, the SF-12 scoring algorithm generates a Physical health Component Summary (PCS-12) score and A Mental health Component Summary (MCS-12) score. These scores are created by weighting and then summing the SF-12 item responses using two separate sets of weights (a physical weight and a mental weight) and then by normalizing the weighted sums to be comparable with a population mean score of 50 with a standard deviation of 10 [23]. The lower the physical health score (PCS-12) or mental health score (MCS-12), the more activity limitations a person has.

TB treatment status, HIV status and antiretroviral treatment was assessed by self-report and from medical information. HIV testing status was assessed by self-report. Anti TB medication adherence was assessed with the question “In your tuberculosis treatment in the past 3–4 weeks how many percent were you taking your anti-tuberculosis medication?” using a Visual Analogue Scale (VAS). TB medication non-adherence was defined as having taken less than 90 percent their anti-tuberculosis medication. ART adherence was assessed with the question “How many percent of your HIV medication did to take in the past 4 weeks?” using a Visual Analogue Scale (VAS). ART non-adherence was defined as having taken less than 90 percent of ART. Patients were also asked about a list of 10 chronic illness conditions they had been diagnosed with including hypertension, diabetes, depression, stomach ulcer, migraine headache, cancer, arthritis, asthma, diabetes, cholesterol.

### Data analysis

Data were analyzed using Statistical Package for the Social Sciences (SPSS) for Windows software application programme version 19.0. Frequencies, means, standard deviations, were calculated to describe the sample. Data were checked for normality distribution and outliers. For non-normal distribution non-parametric tests were used. Associations of PCS and MCS were identified using logistic regression analyses. By taking the median of the dependent variables (PCS and MCS) as a cut-off point, PCS and MCS were dichotomized as poor = 0 or good = 1. Following each univariate regression, multivariable logistic regression models were constructed. Independent variables from the univariate analyses were entered into the multivariable model if significant at P < 0.05 level. For each model, the R^2^ are presented to describe the amount of variance explained by the multivariable model. Probability below 0.05 was regarded as statistically significant.

## Results

### Social demographic characteristics

The total sample comprised of 4935 participants. Thirty-five (35) participants (0.7%) refused to take part in the study. The final sample, therefore, included 4900 participants of which 54.5% are males and 45.5% are females. The mean age of the participants is 36.2 years (SD = 11.5), with an age range of 18 to 93 years, and 65.2% were between 25 to 44 years old. The majority (72.7%) was never married, 27.7% had completed secondary education, and 17% scored high on the poverty index. From the total sample 76.6% were new TB patients and 23.4% were re-treatment TB patients. From those who had tested for HIV, 76.3% had been tested within the last year and another 16.4% within the last three years, 59.9% were HIV positive, 22.1% of the HIV positive patients were on antiretroviral therapy. Twenty percent reported tobacco use daily or almost daily, and 23.3% were hazardous or harmful alcohol users (AUDIT 8 or more). Regarding adherence to TB medication, 61.1% indicated that they had not missed at least on one day in the past 10 days their medication. From those who were on antiretroviral treatment, 58.2% reported that they had not at least once missed their ARVs in the last seven days. A large proportion (81%) reported psychological distress and 16.3% had been diagnosed with at least one chronic illness condition (see Table 1).

**Table 1:** Socio-demographics and health variables sample characteristics

	**Total**		**Men**		**Women**		**P**
	**(n = 4900)**		**(n = 2671) (54.5%)**		**(n = 2229) (45.5%)**		
	**N**	**%**	**N**	**%**	**N**	**%**	
***Age*** (range 18–93)	36.2	11.5	37.2	11.5	34.8	11.4	0.000
18–24	643	13.3	276	10.6	358	16.5	
25–34	1841	38.1	928	35.7	899	41.4	
35–44	13	27.1	780	30.0	515	23.7	
45–54	671	13.9	399	15.3	259	11.9	
55–64	265	5.5	161	6.2	95	4.4	
65 or more	104	2.2	58	2.2	45	2.1	
***Marital status***							
Never married	3323	72.7	1734	70.2	1589	75.6	0.000
Married/cohabitating	982	21.5	594	24.1	388	18.5	0.000
Separated/divorced/widowed	265	5.8	141	5.7	124	5.9	0.783
***Education***							
Grade 7 or less	1269	26.3	745	28.8	502	23.2	0.000
Grade 8–11	2213	45.9	1126	47.4	960	44.3	0.031
Grade 12 or more	1336	27.7	613	23.7	704	32.5	0.000
***Poverty index (5–20)***							
Low (5)	1592	35.0	882	35.2	710	34.4	
Medium (6–12)	2195	48.2	1117	47.2	1018	49.3	
High (13–20)	768	16.9	433	17.4	335	16.2	
***Geolocality***							
Urban residence	3151	66.2	1691	65.4	1460	67.2	0.212
Rural residence	877	18.4	480	18.6	397	18.3	0.780
Informal settlement	730	15.3	413	16.0	317	14.6	0.181
***Health variables***							
New TB patient	3650	76.6	1946	75.2	1704	78.4	
Retreatment TB patient	1113	23.4	643	24.8	470	21.6	
HIV positive	2585	59.9	1222	53.4	1363	67.4	
HIV negative	1728	40.1	1068	46.6	660	32.6	
Other chronic illnesses							
Zero	3042	72.7	1681	75.1	1361	70.0	0.001
One	680	16.3	335	15.0	345	17.7	
Two	293	7.0	151	6.7	142	7.3	
Three or more	167	4.0	71	3.2	96	4.9	
Partner HIV positive	1192	27.2	600	25.0	592	29.8	
Partner HIV negative or unknown status	3194	72.8	1800	75.0	1394	70.2	
Tobacco use (daily or almost daily)	964	20.0	777	29.5	187	8.5	0.000
Hazardous or harmful alcohol use	1120	23.3	820	31.8	280	13.0	0.000
Psychological distress (K >15)	3913	81.1	2127	80.8	1786	81.4	0.245
Had sex in the past 3 months	2318	51.3	1336	54.1	982	48.0	0.000
On antiretroviral therapy	899	22.1	401	18.8	498	25.8	0.000
Adherence to TB treatment	2191	61.1	1153	64.1	1038	68.6	0.007
Adherence to ART	561	58.2	261	52.6	300	64.1	0.000

### Comparison of SF-12 health survey scale means

The overall physical and mental health scores were 42.5 and 40.7, respectively. Emotional role, general health and bodily pain had the lowest sub-scale scores, while energy and fatigue and mental health had the highest domain scores. Independent Kruskal–Wallis tests found significant positive effects of being TB-HIV co-infected on the domains of mental health functioning, emotional role, energy and fatigue, social function and physical role, while significant negative effects were observed on general health, bodily pain and physical function (see Table 2).

**Table 2:** SF-12 health survey scale means for patients with active TB infected with HIV and without HIV

**SF-12 Subscales**	**TB-HIV infection**	**TB without HIV**	**P-value**	**Total sample**
	**M (SD)**	**M (SD)**		**M (SD)**
General health (GH)	35.5 (12.6)	42.6 (13.4)	0.000	38.5 (13.4)
Bodily Pain (BP)	38.5 (11.8)	40.2 (11.8)	0.000	39.2 (11.9)
Physical Function (PF)	40.5 (11.2)	41.4 (10.7)	0.027	40.9 (11.1)
Physical Role (RP)	40.9 (9.2)	39.8 (9.6)	0.000	40.5 (9.4)
Social Function (SF)	41.0 (11.7)	39.3 (13.0)	0.000	40.5 (12.2)
Mental Health (MH)	43.1 (9.5)	40.6 (10.5)	0.000	42.2 (10.0)
Energy and Fatigue (Vitality) (VT)	49.8 (10.7)	46.3 (11.3)	0.000	48.3 (11.1)
Emotional role (RE)	37.6 (11.1)	36.3 (12.1)	0.000	37.3 (11.5)
**SF-12**				
Physical Health (PCS)	39.6 (9.2)	42.5 (8.4)	0.000	40.7 (9.1)
Mental Health (MCS)	43.7 (9.7)	40.4 (11.4)	0.000	42.5 (10.5)

### Predictors of physical health (PCS)

In multivariable analysis higher educational level (Grade 12 or more) (OR = 1.70, 95% CI = 1.01–2.85), lower psychological distress (OR = 0.27, 95% CI = 0.14–0.52), diagnosed with less than three or more chronic conditions (OR = 0.36, 95% CI = 0.13–0.99) and being HIV negative (OR = 0.25, 95% CI = 0.11–0.61) were significantly positively associated with PCS (see Table 3).

**Table 3:** Predictors of Physical Health (PCS)

	**Unadjusted Odds Ratio (95% CI)**	**Adjusted Odds Ratio (95% CI)**^**a,b**^
***Sociodemographics***		
*Age*	0.98 (0.97–0.99)***	0.99 (0.97–1.02)
Female	1.00	1.00
Male	0.94 (0.83–1.07)	0.85 (0.55–1.31)
*Education*		
Grade 7 or less	1.00	1.00
Grade 8–11	1.41 (1.21–1.65)***	1.08 (0.53–1.99)
Grade 12 or more	1.70 (1.43–2.01)***	1.70 (1.01–2.85)*
*Marital status*		
Never married	1.00	1.00
Married/cohabitating	0.92 (0.79–1.07)	0.91 (0.52–1.59)
Divorced/separated/widowed	0.73 (0.56–0.95)*	0.74 (0.35–1.58)
*Poverty index*		
Low	1.00	1.00
Medium	0.77 (0.67–0.89)***	0.84 (0.52–1.38)
High	1.43 (1.17–1.74)***	1.10 (0.55–2.20)
***Health variables***		
New TB patient	1.00	1.00
TB retreatment patient	0.78 (0.68–0.90)***	0.70 (0.45–1.09)
HIV negative	1.00	1.00
HIV positive	0.55 (0.48–0.63)***	0.25 (0.11–0.61)**
*Chronic conditions*		
Zero	1.00	1.00
One	0.92 (0.77–1.10)	0.63 (0.36–1.13)
Two	0.85 (0.65–1.10)	0.88 (0.41–1.91)
Three or more	0.65 (0.46–0.91)*	0.36 (0.13–0.99)*
Psychological distress (K > 15)	0.37 (0.31–0.44)***	0.27 (0.14–0.52)***
Alcohol: low risk (AUDIT 0–7)	1.00	
Medium (AUDIT 8–19)	0.99 (0.83–1.07)	
High (AUDIT 20–40)	0.83 (0.65–1.06)	
Tobacco use (daily or almost daily)	1.07 (0.92–1.26)	---
Had sex in past 3 months	1.50 1.32–1.71)***	1.38 (0.89–2.14)
On ART	0.80 (0.68–0.94)**	0.56 (0.27–1.13)
TB medication adherence	0.63 (0.54–0.74)***	0.76 (0.46–1.25)
ART adherence	0.41 (0.30–0.56)***	0.79 (0.41–1.52)
Partner HIV negative/unknown	1.00	1.00
Partner HIV positive	0.71 (0.62–0.82)***	0.99 (0.63–1.56)

^a^ Using “enter” LR selection of variables.

^b^ Hosmer and Lemeshow Chi-square 6.10, df 8, 0.637; Cox and Snell R^2^ 0.18; Nagelkerke R^2^ 0.25.

* P < 0.05; ** P < 0.01; *** P < 0.001.

### Predictors of mental health (MCS)

In multivariable analysis low poverty (OR = 0.48, 95% CI = 0.26–0.90), scoring low on the psychological distress scale (OR = 0.09, 95% CI = 0.05–0.17) and being HIV positive (OR = 2.99, 95% CI = 1.61–5.57) were positively significantly associated with MCS (see Table 4).

**Table 4:** Predictors of Mental Health (MCS)

	**Unadjusted Odds Ratio (95% CI)**	**Adjusted Odds Ratio (95% CI)**^**a,b**^
***Sociodemographics***		
*Age*	1.00 (0.99–1.00)	1.02 (1.00–1.03)
*Gender*		
Female	1.00	1.00
Male	1.11 (0.98–1.00)	1.21 (0.83–1.77)
*Education*		
Grade 7 or less	1.00	1.00
Grade 8–11	1.16 (0.99–1.35)	0.75 (0.48–1.17)
Grade 12 or more	1.42 (1.20–1.68)***	1.02 (0.59–1.74)
*Marital status*		
Never married	1.00	
Married/cohabitating	1.13 (0.97–1.32)	
Divorced/separated/widowed	0.80 (0.61–1.04)	
*Poverty index*		
Low	1.00	1.00
Medium	0.98 (0.85–1.13)	0.98 (0.65–1.48)
High	0.31 (0.25–0.38)***	0.48 (0.26–0.90)*
***Health variables***		
New TB patient	1.00	
TB retreatment patient	0.93 (0.80–1.07)	
HIV negative	1.00	1.00
HIV positive	1.58 (1.38–1.80)***	2.99 (1.61–5.57)***
*Chronic conditions*		
Zero	1.00	
One	1.05 (0.87–1.26)	
Two	1.20 (0.93–1.56)	
Three or more	1.13 (0.80–1.59)	
Psychological distress (K > 15)	0.28 (0.23–0.33)***	0.09 (0.05–0.17)***
Alcohol: low risk (AUDIT 0–7)	1.00	
Medium (AUDIT 8–19)	0.92 (0.78–1.09)	
High (AUDIT 20–40)	1.00 (0.78–1.27)	
Tobacco use (daily or almost daily)	0.81 (0.69–0.94)**	0.85 (0.50–1.45)
Had sex in past 3 months	0.75 (0.66–0.85)***	0.77 (0.53–1.11)
On ART	1.05 (0.89–1.23)	---
TB medication adherence	1.11 (0.95–1.30)	---
ART adherence	1.98 (1.48–2.67)***	1.19 (0.78–1.87)
Partner HIV negative/unknown	1.00	1.00
Partner HIV positive	1.31 (1.13–1.51)***	1.45 (0.99–2.16)

^a^ Using “enter” LR selection of variables.

^b^ Hosmer and Lemeshow Chi-square 14.39 df 8, 0.072; Cox and Snell R^2^ 0.21; Nagelkerke R^2^ 0.27.

* P < 0.05; ** P < 0.01; *** P < 0.001.

## Discussion

The study found among a large sample of tuberculosis public primary care patients in South Africa overall low physical and mental health QoL scores (42.5 and 40.7 on the SF-12, respectively) at the beginning of TB treatment. This finding seems significantly lower than found in TB patients in China [6] and among people living with HIV and AIDS [5]. Further, in the present study significant positive effects of being TB-HIV co-infected on mental health QoL and significant negative effects were observed on physical function QoL. In contrast with the present study, Deribew’s [1] study included HIV positive individuals with and without TB. They found lower mean scores on all domains for people co-infected with HIV and TB compared to people living with HIV without TB. Overall, according to a systematic review done in 2009, findings indicate that anti-TB treatment had a positive effect on improving health-related QoL, more so on the physical health of patients than the mental health [24]. So it could be that physical health improves with anti-TB medication greater in TB only patients than in TB-HIV co-infected patients. Kittikraisak et al. [8] found improved health utility after TB treatment was more evident in HIV-infected patients than those uninfected in almost all domains. This they report may be a result of the relief of some TB symptoms and adverse events from TB and HIV drug interactions [8] or after dealing with the effect of HIV disease severity [3].

In multivariable analysis higher educational, lower psychological distress, having fewer chronic conditions and being HIV negative were significantly positively associated with physical health QoL, and low poverty, low psychological distress and being HIV positive were positively significantly associated with mental health QoL. Other studies [1,10] also found that low psychological distress (depression) was associated with higher QoL in TB patients. Being diagnosed with several chronic conditions and scoring high on the psychological distress scale, may further increase levels of anxiety and depression given the rigorous treatment regimen. The finding that indicators of higher socio-economic status (education, low poverty) in this study were associated with better QoL in TB patients is in agreement with a number of studies [1,8,25]. Unlike in some other studies [3,4,26-29], this study did not find age and gender differences in relation to health-related QoL in TB patients.

### Study limitations

This study has several limitations. Caution should be taken when interpreting the results of this study because of certain limitations. Although the Zulu version of the SF-12 instrument was used previously in South Africa, the content and criterion validity of the instrument was not assessed. However, we found good correlation between the MCS and the Kessler Scale (correlation coefficient, r = −0.34, P = 0.001). As this was a cross-sectional study, causality between the compared variables cannot be concluded. A further limitation was that most variables were assessed by self-report and desirable responses may have been given. Also verification of the presence of chronic illnesses by history alone could introduce bias. Data collected on CD4 counts and WHO staging were incomplete in this study and were therefore not included in the analysis, although this would have been important as they could have been related to QoL.

## Conclusion

TB and HIV cause deterioration in patients’ health-related quality of life. Even though progress in scaling up interventions to address the co-epidemics of TB and HIV has continued [30,31], new ways to strengthen infection control is needed. Thus, it is imperative that TB control programmes at public health clinics design strategies to improve the quality of health of TB and/or HIV co-infected patients.

## Competing interests

The authors declare that they have no competing interests.

## Authors’ contributions

JL, KP, PN and GM were the main contributors to the conceptualization of the study. JL, KP contributed significantly to the first draft of the paper and all authors contributed to the subsequent drafts and finalization. All authors read and approved the final manuscript.
